# Recurrent American Cutaneous Leishmaniasis

**DOI:** 10.3201/eid1309.061446

**Published:** 2007-09

**Authors:** Jean-Pierre Gangneux, Sylvie Sauzet, Sébastien Donnard, Nicolas Meyer, Anne Cornillet, Francine Pratlong, Claude Guiguen

**Affiliations:** * Centre Hospitalier Universitaire Faculté de Médecine de Rennes, Rennes, France; † Service Medical du 11ème Régiment d'Artillerie de Marine, Saint-Aubin du Cormier, France; ‡ Université de Montpellier, Montpellier, France

**Keywords:** Cutaneous leishmaniasis, Leishmania guyanensis, pentamidine, leishmaniasis recidiva cutis, leishmaniasis recidivans, recurrence, letter

**To the Editor:** Leishmaniasis recidivans is an unusual clinical Old World disease primarily associated with *Leishmania tropica* (*1*). Recurrence of previously cured cutaneous leishmaniasis (CL) lesions is found in American CL, for which a specific nosologic form known of disease known as leishmaniasis recidiva cutis (LRC) has been identified. Although <30 cases of LRC have been reported from Brazil, Colombia, Peru and Ecuador; these cases were caused mainly by *L*. *braziliensis*, *L*. *amazonensis*, and *L*. *panamensis* (*2*). We report 7 cases of recurrent American CL caused by *L*. *guyanensis* in French Guiana.

Forty-eight military personnel who lived in France spent 3 months in French Guiana in 2004 and took part in a military training program in the rainforest for 15 days. Despite similar exposure conditions, American CL, confirmed by positive direct examination of Giemsa-stained tissue smears, developed in 21 persons. These patients were treated with 1 or 2 courses of either 3 intravenous or 2 intramuscular injections of pentamidine isethionate (4 mg/kg on alternate days). All lesions were cured 1–3 months after treatment had ended. Recurrence of the CL lesion was observed in 7 patients after a disease-free interval of 3–6 months (Table).

**Table:** Epidemiologic characteristics, strain identification, and treatment of 21 cases of American cutaneous leishmaniasis, French Guiana, 2004–2005*

Case no.	Primary infection					Leishmaniasis recidiva cutis			
	Lesion(s), location (no.)	PI treatment†	Time to healed lesions, mo	Disease-free interval, mo		Direct examination	Strain identification	PI treatment	Outcome
1	Thorax (1)	IV, 4 mg/kg on alternate days × 3	1	4		+	*Leishmania guyanensis* MON-45	IV, 4 mg/kg on alternate days × 4	Cured
2	Right forearm (1)	IV, 4 mg/kg on alternate days × 3	3	6		+	*L. guyanensis* MON-45	IV, 4 mg/kg on alternate days × 4	Cured
3	Left leg (1) and chin (3)	IV, 4 mg/kg on alternate days × 3	1	4		+	Negative culture	IV, 4 mg/kg on alternate days × 4	Cured
4	Chin (3)	IV, 4 mg/kg on alternate days × 3	1	4		+	Negative culture	IV, 4 mg/kg on alternate days × 4	Cured
5	Behind left ear (1)	IV, 4 mg/kg on alternate days × 3	1	3		+	*L. guyanensis* MON-131	IV, 4 mg/kg on alternate days × 4	Cured
6	Left ear (1)	IV, 4 mg/kg on alternate days × 3	1	6		+	Negative culture	IV, 4 mg/kg on alternate days × 4	Cured
7	Right ankle (5)	IV, 4 mg/kg on alternate days × 3	2.5	4		+	Negative culture	IV, 4 mg/kg on alternate days × 4	Cured
8–21	Various	IV, 4 mg/kg on alternate days × 3 (for 9 patients) or IM, 4 mg/kg on alternate days × 2 (for 5 patients)†	1–3	–		–	–	–	–

*PI, pentamidine isethionate; IV, intravenous; IM, intramuscular. †IV versus IM administration of pentamidine isethionate was not associated with recurrent forms.

New lesions appeared on the edge of a healed scar for each patient, regardless of the location of the primary lesion (Table), and were diagnosed at Rennes University Hospital (positive direct examination or culture) in 2005. For positive cultures, *L*. *guyanensis* was identified by genomic and isoenzymatic characterization at the Centre National de Référence des *Leishmania*, Université de Montpellier, Montpellier, France. Patients were treated with 4 intravenous injections of pentamidine isethionate (4 mg/kg every other day) and were cured without recurrence within 2 years. No differences in age or underlying diseases were noted in patients with recurrent CL.

*L*. (*Viannia*) *guyanensis* is highly prevalent in several leishmaniasis-endemic areas of Brazil, Colombia, French Guiana, Guyana, Surinam, Peru, and Ecuador. This organism accounts for >95% of the 5 *Leishmania* species found in French Guiana, commonly causes localized CL, and occasionally causes disseminated CL and mucocutaneous leishmaniasis (*3*). Dedet et al. reported that 6.8% of patients with CL caused by *L*. *guyanensis* had a recurrent lesion at the site of a previously cured lesion, which occurred after a mean interval of 7.3 months (*4*). A total of 33% of our patients had a cured primary infection in <3 months but they had a recurrence after a disease-free interval 3–6 months after treatment.

Additional information on such a recurrent form of CL is needed. Clinical symptoms in our patients were suggestive of LRC as described by Berlin (*1*)*,* i.e., a recurrence at the site of an original ulcer, generally within 2 years and often on the edge of a scar. LRC may not be uncommon in the New World but rather underreported (*2*). Few cases of LRC have been reported; these were caused by *L*. *braziliensis*, *L*. *amazonensis*, and *L*. *panamensis* (*2*,*5*,*6*). In CL caused by *L*. *guyanensis*, borderline clinical symptoms prevent clear distinction of the recurrent form of LRC from early treatment failures or reinfections. In our patients, the risk for reinfection was excluded because the military personnel lived in France and left French Guiana several months before the recurrence.

Although pentavalent antimony is the recommended treatment for American CL, pentamidine isethionate is widely used in French Guiana. Retrospective analysis showed that 5%–25% of early treatment failures occurred after 1 or 2 intramuscular injections of 4–7 mg/kg of pentamidine isethionate, depending on different risk factors (*7**,**8*). Our observational study was not designed to evaluate treatment efficiency; we observed 2 (9.5%) of 21 early relapses and 7 (33%) of 21 late-onset recurrences in this series. Although we lacked statistical power because of small numbers, a difference was observed in recurrence by treatment method in 7 (44%) of 16 with recurrent disease who received 3 intravenous pentamidine isethionate injections compared with 0 (0%) of 5 who received 2 intramuscular injections. Re-treatment with a regimen of 4 intravenous injections of pentamidine, under medical surveillance because of possible adverse effects, cured the disease. Physicians in countries in which *L*. *guyanensis* is endemic should be aware of these complicated forms of CL after treatment, forms that prompt long-term follow-up and evaluation of specific therapeutic protocols.

Finally, the mechanism of late-recurring leishmaniasis is poorly understood. Mendonça et al. suggested that clinical cure of American CL is rarely associated with sterile cure (i.e., elimination of the parasite) (*9*). Immunologic data based on skin hypersensitivity and histopathologic and immunohistochemical findings support the concept that LRC is a late-onset reactivation after persistence of living parasites around or in cured leishmaniasis by as-yet unknown stimuli such as local trauma (2 of our patients reported chronic lesions of the chin caused by a razor blade and 2 others had chronically scratched the scar lesion on their ears) or corticosteroid, and after an incomplete host immune response to an earlier episode (*2**, **5**,**10*). Further immunologic studies of patients and identification of genetic characteristics of *L*. *guyanensis* are needed to address this question.
